# Consumers of natural health products: natural-born pharmacovigilantes?

**DOI:** 10.1186/1472-6882-10-8

**Published:** 2010-02-25

**Authors:** Rishma Walji, Heather Boon, Joanne Barnes, Zubin Austin, Sandy Welsh, G Ross Baker

**Affiliations:** 1 Department of Pharmaceutical Sciences, Leslie Dan Faculty of Pharmacy, University of Toronto, Toronto, Canada; 2 School of Pharmacy, Faculty of Medical and Health Sciences, University of Auckland, Aukland, New Zealand; 3 Department of Sociology, University of Toronto, Toronto, Canada; 4 Faculty of Medicine, University of Toronto, Toronto, Canada

## Abstract

**Background:**

Natural health products (NHPs), such as herbal medicines and vitamins, are widely available over-the-counter and are often purchased by consumers without advice from a healthcare provider. This study examined how consumers respond when they believe they have experienced NHP-related adverse drug reactions (ADRs) in order to determine how to improve current safety monitoring strategies.

**Methods:**

Qualitative semi-structured interviews were conducted with twelve consumers who had experienced a self-identified NHP-related ADR. Key emergent themes were identified and coded using content analysis techniques.

**Results:**

Consumers were generally not comfortable enough with their conventional health care providers to discuss their NHP-related ADRs. Consumers reported being more comfortable discussing NHP-related ADRs with personnel from health food stores, friends or family with whom they had developed trusted relationships. No one reported their suspected ADR to Health Canada and most did not know this was possible.

**Conclusion:**

Consumers generally did not report their suspected NHP-related ADRs to healthcare providers or to Health Canada. Passive reporting systems for collecting information on NHP-related ADRs cannot be effective if consumers who experience NHP-related ADRs do not report their experiences. Healthcare providers, health food store personnel, manufacturers and other stakeholders also need to take responsibility for reporting ADRs in order to improve current pharmacovigilance of NHPs.

## Background

Despite the belief that herbal medicine and other natural health products (NHPs) are safe [1], these products are pharmacologically active and therefore have inherent risk. Under Canadian federal regulations, NHPs are technically a sub-category of drugs. Any substance naturally found in plants, animals, fungi, algae or micro-organisms (regardless of the source used for the supplement) that is used to diagnose, treat or prevent disease and is suitable for self-care use is categorized as a NHP in Canada. This category includes vitamins (regardless of source), minerals, traditional Chinese medicines, Ayurvedic medicines, Native North American medicines, traditional herbal remedies and homeopathic medicines. Biologics such as insulin, tobacco and marijuana are specifically excluded from the NHP definition [2]. Several reviews clearly document that NHPs, especially herbal medicines, are associated with adverse drug reactions (ADRs) (See Table 1: ADR Definitions) [3,4]. The need to understand ADRs associated with NHPs is increasingly important, given that over 60% of all North Americans report using some form of complementary and alternative medicine (CAM) (including herbal medicines) in the management of their health [1,5]. In Canada, 7 in 10 adults have ever used an NHP [1].

**Table 1:** ADR Definitions

Term	Definition
Adverse Drug Reaction (ADR)	A noxious and unintended response to a drug, and which occurs at doses normally used in persons for the prophylaxis, diagnosis, or therapy of disease, or for the modification of physiological function.

Suspected ADR	An adverse event that for which there is a suspicion of a causal relationship with a drug

Adverse event/experience	Any untoward medical occurrence that may present during treatment with a pharmaceutical product but which does not necessarily have a causal relationship with this treatment.

Serious ADR	Fatal, life threatening (such as liver failure, abnormal heart rhythms, certain types of allergic reactions), result in persistent or significant disability or incapacity, require or prolong hospitalization, are congenital anomalies or birth defects, or are otherwise medically important.

Severe ADR	Severity describes the intensity of the adverse event or ADR. A severe ADR (e.g., a severe headache) is not necessarily a serious ADR

• World Health Organization Upsalla Monitoring Centre: The Global Intelligence Network for Benefits and Risk in Medicinal Products http://www.who-umc.org/ Accessed on: Feb 8, 2010

• World Health Organization: Note for guidance on clinical safety data management: definitions and standards for expedited reporting. 1995.

Although NHPs are commonly used, relatively little is known about the frequency of NHP-related suspected ADRs. In Canada, Health Canada collects reports of suspected ADRs from healthcare professionals, and from consumers, about NHPs (and conventional drugs) through the Canada Vigilance Program [6]. Similar systems exist in many developed and developing countries, and the World Health Organization's Collaborating Centre for International Drug Monitoring co-ordinates global ADR data and searches for signals of safety concerns. Under-reporting is a challenge of such passive reporting systems [7]. Notwithstanding the problems in this arena for pharmaceuticals, there is at least a well known avenue for reporting and feedback loops to practitioners and, to some extent, consumers. However, experiences of (and the incidence of) herbal medicine-related ADRs are more difficult to determine because under-reporting of suspected ADRs, may be more substantial for NHPs than for conventional drugs [8].

One of the reasons for under-reporting of NHP-related ADRs may be that many consumers believe NHPs are safe because they originate from natural ingredients [1]. Consumers frequently self-prescribe NHPs, without the advice of a qualified health provider [9]. Increasing consumerism, an environment in which individuals are taking their health matters into their own hands, partly explains this [10]. Instead of passively following the advice of health providers, individuals explore opinions and advice from a range of information sources (e.g., such as the internet, friends and family) and increasingly challenge healthcare practitioners with questions about treatments [11].

In Canada, NHPs are regulated as a sub-category of drugs. By definition, they are available over-the-counter and include such products as herbal medicines, vitamins and homeopathic preparations. Since NHPs are non-prescription medicines, in order to capture safety-related information NHP-related ADRs must be reported by consumers: consumers must either inform healthcare professionals, who are then encouraged to file an official report, or consumers may directly inform Health Canada. If these steps are not taken, the ADR is not likely to be captured by the national reporting system. Since many consumers self-prescribe NHPs, it is perhaps not surprising that consumers appear less likely to report ADRs associated with NHPs to their healthcare providers than those associated with conventional over-the-counter drugs [12]. Even where consumers inform healthcare professionals of suspected ADRs associated with NHPs, these reports may not be filed with the Canadian Vigilance Program (Health Canada's ADR reporting system) [13].

If natural health products have potential deleterious effects both on their own and in combination with other drugs, then they also need to be monitored. Yet the current systems are ineffective. The purpose of this study was to explore how users of NHPs identify and respond to NHP-related suspected ADRs. Specifically, the study examined the experiences of consumers who had experienced what they believed to be an NHP-related ADR and their reasons for choosing to report (or not report) their reactions. This information is crucial in order to better understand why NHP-related ADRs are so rarely reported.

## Methods

This was an exploratory descriptive qualitative study. Qualitative methods were best suited for this study because although under-reporting is a well-known weakness of passive surveillance systems, it is not at all clear how consumers react when they believe they have experienced an ADR or why they do what they do. Additionally, NHPs constitute a highly contextualized category of medicines, in that they have their own unique classification and perceptions associated with them. The methods chosen allowed exploration of the idiosyncrasies associated with this topic. In-depth semi-structured interviews were conducted from September 2007 to December 2007 with NHP consumers who had experienced a self-identified suspected ADR associated with use of an NHP within the past 5 years. Inclusion criteria for participants were: have experienced a suspected ADR that they perceived to be associated with an NHP in the past 5 years; able to participate in an interview in English; gave informed consent to participate. While we had no way to determine if an ADR had actually occurred, the participant's perception of an NHP-related ADR was the key inclusion criterion because the purpose of the study was to examining their experiences and behaviours in response to this perceived event.

The participants were recruited primarily from the Greater Toronto area. Some consumers also volunteered to participate from Ottawa and Vancouver as they became aware of the study from their social networks. Participants were recruited through posters and advertisements in several university email listservs, online networking groups such as facebook, health food stores, pharmacies and hospital lobbies. Recruitment began with a convenience sample of individuals referred by the study team's extensive professional and social networks. In addition, snowball sampling (in which participants were asked to identify other potential participants within their social circles) was employed to extend the sample [14]. The recruitment ads requested that anyone who had experienced what s/he believed to be an NHP-related ADR call the study centre. Callers were screened to ensure they met the inclusion criteria. Every effort was made to recruit a maximum variation sample with a range of sociodemographic backgrounds by placing recruitment materials in a wide range of locations; however, given the difficulty we encountered in finding people that identified experiencing a NHP-related ADR, all those who met the inclusion criteria for the study were interviewed.

All interviews were conducted by RW after written informed consent was obtained. Interviews were based on a semi-structured interview guide derived from the collective experience of the authors and a review of the literature (see Table 2: Questions for Consumer Guided Interview). Interviews lasted approximately 30-60 minutes and took place at a private location most convenient to the participant (e.g., participant's home, in the researcher's office). All interviews were audio-recorded so that the verbatim transcriptions and field notes could be coded. Field notes were hand-written during and immediately following the interviews.

**Table 2:** Questions for Consumer Guided Interview

1. What prompted you to take this natural health product?
2. Where did you purchase your product? (e.g. Health food store? Pharmacy? Internet? Other?)
3. Is this where you usually buy your natural health products? Why do you buy them from there?
4. Can you describe what (side effect) happened to you? How soon after you took the product did it happen? How severe was it? Exactly what symptoms did you experience?
5. What did you do about it? Why?
a. Did you tell anyone (and why or why not)? If your side effects were more mild/moderate/severe, would you have responded differently?
6. Do you think that people should report their side effects? Why or why not?
a. Where would be an appropriate place to report? Medical Doctor, Pharmacist, Naturopath, Retail store where they bought it, Manufacturer, Media, Other.
7. What kinds of side effects do you think should be reported?
8. What would prompt you to report (or not report) a side effect?
9. What do you think about the safety of natural health products in general?
a. How safe did you think the product you took was before you used it?
10. Where do you get information about natural health products? Why that source?
11. Are you familiar with the reporting system for adverse drug reactions in Canada (describe)? What do you know about it? Have you ever used it?
What for? (e.g. To send in a report? Or to look at the reports that other people have made?)
12. What do you think are the major obstacles in reporting side effects from a natural health product?
13. Are you satisfied with the current procedure of adverse event reporting system for natural health products? Why or why not?
a. Has Health Canada and/or your health practitioner provided any information, assistance, or feedback to you about reporting procedures?
b. What can be done to enable reporting of side effects from natural health products?

Two independent coders used a constant comparison method of content analysis to identify key themes in the interviews [15]. Disagreement was resolved through in-depth discussion. A software program, NVIVO 7, was used to organize and apply coding to the data [16]. Data analysis and coding took place throughout the process of data collection. The interview guide was updated and modified after the first couple of coding sessions to allow for increasingly detailed data collection in key emerging themes.

Interviewing continued until theoretical saturation of the key themes was achieved [14]. Saturation is defined as the point at which no new information or themes emerge from the data [14,17]. In this study, saturation was identified in themes related to why people did or did not tell anyone about the NHP-related suspected ADR after approximately 10 interviews. Two additional interviews were completed to confirm that saturation had been achieved.

Ethics approval for this study was granted by the Research Ethics Board at the University of Toronto. All personal identifiers have been removed or disguised so the person(s) described are not identifiable.

## Results

Participants varied in age and demographic background (see Table 3: Participant Demographics) and had experienced a range of different NHP-related suspected ADRs such as rash, nausea, digestive disturbance and anxiety/irritability. The final sample was predominantly female and highly educated, although efforts were made to recruit more males and participants from different socioeconomic backgrounds. All the suspected ADRs experienced by the participants in this study were non-serious (i.e., did not require hospitalization or lead to permanent disability or death), but differed in perceived degree of severity from mild to moderate to severe (see Table 4: Participant Reactions and Behaviours).

**Table 3:** Participant Demographics

ID #	Age	Sex	Education	Experience with NHPs^1^
1	25	F	Post-secondary	Work at HFS
2	28	F	Health care professional	NHP training
3	43	M	High school	None
4	24	F	Post-secondary	None
5	31	F	Post-secondary	None
6	29	F	Post secondary	None
7	42	F	Post- secondary	None
8	36	F	Health professional	NHP training
9	38	F	High school	None
10	28	F	Post- secondary	Worked at HFS
11	22	F	High school	None
12	34	F	Post- secondary	None

^1^Experience with NHPs was only described if it was beyond a lay level of knowledge.

**Table 4:** Participant Reactions and Behaviours

ID #	Product	Reason for taking NHP	Self prescribed	**ADR **^**1**^	**Severity **^**1,2**^	Outcome	Reporting Behaviour
1	Stress relief supplement (ingredients include L-theanine)	For alertness/Stress	Self prescribed	Rash	Moderate	Recovered with OTC treatment	Reported to PHM, manufacturer via HFS

2	Fish oil	For health maintenance	Self prescribed	Rash	Mild	Recovered after d/c	Reported to CAM

3	Weight loss product ingredients unknown)	For weight loss	Self prescribed	Stimulation, irritability, road rage	Severe	Recovered after d/c	Report to f/f, HFS

4	Digestive enzymes (ingredients include amylase, protease, lipase)	For digestive concerns	Self prescribed	Stomach pain, bloating, heartburn	Moderate	Re-challenged, Recovered after d/c	Did not report

5	1. Herbal cleanse 2. [Wished not to disclose product type or name]	1. Cleansing product 2. For energy	1. Self prescribed 2. Self prescribed	1. flatulence, bowel urgency, stomach discomfort 2. anxiety, depression, nervousness, nausea	1. moderate to severe 2. moderate to severe	1. recovered after d/c 2. recovered after d/c	1. Did not report 2. Contacted manufacturer

6	1. *Ginkgo biloba* 2. weight loss product 3. St. John's wort	1. for memory 2. for weight loss 3. for mood	1. Self prescribed 2. Self prescribed 3. Self prescribed	1. sweating 2. shaky, disoriented 3. photosensitivity	1. mild 2. severe 3. mild	1. recovered after d/c 2. recovered after d/c 3. recovered after d/c	1. Did not report 2. Did not report 3. Did not report

7	Valerian	For sleep	Self prescribed	Agitated, unsettled	Moderate	Recovered after d/c	Did not report

8	Fish oil	For health maintenance	Self prescribed	Rash	Mild	Recovered after d/c	Report to CAM, f/f, manufacturer

9	Thyroid glandular	For weight loss	MD prescribed	Headache, nausea	Severe	Changed dose	Report to MD

10	Multi-vitamin	For health maintenance	Self prescribed	Nausea, vomiting	Severe	Re-challenged, recovered after d/c	Report to f/f

11	Weight loss product	For weight loss	Self prescribed	Heartburn	Mild to moderate	Re-challenged, recovered after d/c	Report to f/f

12	Melatonin	For sleep	Self prescribed	Groggy, uncoordinated	Mild to moderate	Recovered after d/c	Did not report

^1 ^some participants experienced reactions to more than one product; ^2 ^severity is classified according to patient definition of severity; d/c = discontinuation; CAM = complementary and alternative medicine practitioner; MD = medical doctor; PHM = pharmacist; F/f = friends and family; HFS = health food store

Although Health Canada accepts direct reporting of suspected NHP-related ADRs by consumers, the participants did not seem to know that was an option. The only ones who were aware of their ability to independently report their suspected ADRs were told this by their health providers, or personally had some kind of health care training and, therefore, had been exposed to the reporting scheme through their professions.

The main themes that emerged from this research were: 1) how participants first identified their suspected ADRs; 2) how they associated the reaction with their NHP treatments; 3) what influenced their likelihood of reporting the suspected ADR to health care providers, the Canadian Vigilance Program, or anyone else; 4) barriers and enablers to reporting.

### Identifying the suspected ADR as NHP-related

Once they had experienced ADRs, the consumers first had to identify that they were in fact ADRs and not just new illnesses or worsening of pre-existing conditions. Consumers tended to use a process of elimination, re-challenge and investigative reasoning to evaluate their symptoms.

I was trying to think back to all the things I'd eaten the day before. I took, like maybe two, two additional ones and like within an hour the rash had spread ... and that's when I was like, oh, it must be this new product! (Consumer 1)

I had never experienced anything like that before. (Consumer 6)

There had to be a change within my diet or my lifestyle or something to trigger this headache... I kind of know when something might be different or changing so I knew, I just felt that this migraine was triggered by the medication. I can't see it being anything else. (Consumer 9)

Most participants chose to consume the NHPs that they associated with suspected ADRs without advice or monitoring from a healthcare provider. Thus, when they experienced the suspected ADR, they felt obliged to take responsibility to interpret the reaction on their own. Participants reported methodically evaluating their reactions in order to determine the purported cause. They generally came to the conclusion that the symptoms they were experiencing were related to the NHP after considering the possibility of alternative explanations for the symptoms. For example, they ruled out changes in lifestyle, behaviour or other medication, and also based their decision on criteria such as the temporal proximity of ingestion of the product to the reaction, by trying a different brand of the product and, in some cases, stopping (de-challenge) and then re-starting (re-challenge) the product suspected of causing the reaction:

Question: How did you decide that it was the product that caused these things?

I guess because I'd never taken it before.... that was the only thing I could attribute it to. (Consumer 12)

From using one brand and then switching to another, the fact that they were different brands but they were the same ingredients I was using. They contained the same things. I was just trying another brand and I was still getting the same symptoms and then I also linked it because just at this time when I was taking it, I wasn't taking any other products at that time. (Consumer 4)

I noticed if I skipped it, like I forgot -- it's lunch or something - then I didn't have the symptoms ... so, yah, that was a clue. (Consumer 5)

I would take it and about within an hour, I would start to feel nauseous (Consumer 10)

I think because other people developed it and the only thing we had in common was we took this product. (Consumer 2)

I had some, you know a little bit later. The same thing happened and so I put two and two together. (Consumer 6)

When you're dealing with something that's given for dieting you sort of know that there's a chance that it might be something that it shouldn't be and so I just figured that that was probably something that it wasn't. (Consumer 6)

### Likelihood of reporting

Participants' perceptions of the severity and seriousness of their ADRs appeared to influence their behaviour. Most participants confused the meaning of severe and serious, and used them interchangeably. If they perceived the reactions to be mild and/or non-serious, they would generally attempt to mitigate the symptoms independently. Participants claimed that if their reactions had been severe, they would be more likely to seek help or support with managing NHP-related ADRs:

Question: What would make you more likely to talk to someone about your reaction?

I suppose a cardiac event, like, for sure you would want to tell somebody about that. I think that would be significant. If you broke out into a rash, you should probably tell somebody about that. If a product, you know, made you nauseous or dizzy or something like that, you might want to mention that to somebody as well. (Consumer 7)

If the adverse event is mild then it just won't come up, but if it is severe and it's an emergency situation or if it has ongoing repercussions in health [i.e., serious] then it's vital that they be disclosed. (Consumer 5)

I guess if it was a more severe reaction or if it hadn't cleared up ... [then I'd] go the hospital or something like that. (Consumer 1)

Interestingly, some participants who experienced what they defined as severe ADRs still did not report the reaction to anyone or seek advice. This suggests that there may be a difference between how consumers say they would act, and what they actually did. Hypothetically, more severe or serious reactions would result in an increased likelihood of reports.

### Barriers and enablers to reporting

Participants outlined a range of reasons for not reporting. Barriers to reporting included: taking responsibility for self prescribing, lack of awareness of who to tell and the specific reporting process, perceived complexity of the reporting process and fear of losing access to NHPs. Similarly, the main enablers to reporting were feeling comfortable with the person with whom they shared their ADR experience and knowing that one could or should report the ADR.

Many participants believed that since they had independently made the decision to take NHPs, they also had a responsibility to "deal with" the negative consequences of that decision on their own. Consumers' decisions regarding how to respond to NHP-related ADRs therefore depended largely on whether or not they believed themselves to be responsible for the decision to initiate NHP use without advice from a healthcare practitioner:

We often combine products in a way that it can be detrimental to our health and I believe that this adverse reaction had to do with that more than the product itself... so that just has to do with irresponsible consumption... So yeah, the adverse reaction is my problem. (Consumer 5)

I mean I told my doctor about the [pharmaceutical medication] because he was the person who prescribed it to me. I didn't tell anybody about the [NHP] because nobody prescribed it to me so I thought, well, I guess I didn't think that I should tell anyone. I think I just didn't think I should tell anybody! (Consumer 7)

I didn't [report] because there wasn't anyone really to tell about it because I wasn't seeing a doctor at the time and I was more sort of trying things on my own. (Consumer 10)

A key reason why consumers experiencing NHP-related suspected ADRs did not report was because they did not know who to talk to. In part, this was because they had self-purchased the NHP and didn't think there was anyone to turn to. In some cases, the decision not to discuss their experiences with others was because they didn't feel that they could confide in their health care providers (often physicians). These participants perceived that their health care providers would disapprove of their use of NHPs, or not give adequate attention to their concerns:

After I figured out what was going on I knew it was me doing it to me, by choosing to take it, I knew that that I was the cause of it but my doctor wouldn't do anything about it and she would just kind of blow it off so, there would be no point in taking it to her because I just didn't have a therapeutic relationship with her. (Consumer 10)

Well our family doctor is, sort of, you know, she likes to take things lightly, so in a sense sometimes I feel like I shouldn't even go so I should wait things out before I talk to her about them because I feel like she's just going to say the same thing to me, like, oh well, whatever, just go home and have some rest and it will be okay. It's a matter of, there hasn't been the time, the time has not been permitted to go into much detail about it. (Consumer 7)

These quotes illustrate the challenges that many participants had with their conventional medical practitioners, especially when discussing issues associated with NHPs. Most were of the opinion that their healthcare providers (most often physicians and pharmacists) would not support their decisions to try NHPs and this resulted in a lack of communication about suspected NHP-related ADRs:

Her [the pharmacists'] initial reaction when I told her [that I had had a reaction to an NHP], was like, there's no need for me to discuss this any further. (Consumer 1)

I've had a doctor tell me that I [the doctor] don't really believe in all that [NHP] stuff. So I personally wouldn't bother telling them about my adverse effects. I'd just tell the naturopath, or the health food store. (Consumer 4)

If participants thought their friends and family would understand their decisions to use NHPs, they might share their NHP-related ADR experiences with people in their social network. Other participants, who wanted reassurance or guidance about their experiences, discussed approaching other resources such as retailers at the location where they purchased NHPs such as pharmacists and health food store personnel. However, overall, they seemed not to have strong relationships with their pharmacists, which negatively affected their likelihood of reporting ADRs to pharmacists:

Let's say that I buy something from [drug store name]. If I'm not happy with it, generally if I can't return it. ... I probably wouldn't take it back or take any type of concern to that particular store because I feel like the people that work there would not necessarily not care, but that they just don't want to deal with that kind of thing. They have so many other things that they're selling in that store. It's just not an open and friendly environment to bring stuff to. With regards to smaller stores, smaller health food stores, it's an environment where the owner always tries to have a good customer relationship. (Consumer 10)

In some cases, participants described a general distrust of conventional medicine which appeared to be driving their use of NHPs in the first place and was likely a key factor in why they did not turn to conventional health care providers for help. This also seemed to be related to perceptions of conventional medicine having much higher risk than NHPs which were thought to be safe:

I come from a family with immensely little trust of any sort of medical doctors ... especially conventional medical practitioners so I try avoid medicines in general (Consumer 6)

I think in general natural health products are safer to take. You can take them for a longer period of time, you can take them for many different conditions as well. (Consumer 8)

Participants tended to feel comfortable with their health food store personnel and often described good relationships with the staff working in these retail settings. Consumers reported reliance on the health food store staff for information and feeling comfortable talking to them about side effects or problems with their products:

I would trust [staff] more from a health food store because they do - usually the owners of the store have good information and have some knowledge whereby the pharmacies would have nothing, and a pharmacist would probably bash it down anyway! (Consumer 3)

Besides health food stores, participants also felt comfortable contacting the NHP manufacturer in situations of suspected ADRs.

I contacted [the manufacturer via the health food store]... I just wanted to tell them, in case anyone else had the same thing. That way the more people who talk about it the more chances that they'll actually do something. (Consumer 1)

Because I was satisfied with the product and then something changed [from the last time I used it], and I was extremely unsatisfied with it, I felt the need to address that. (Consumer 5)

I feel like I did have resources and I certainly followed up on them [with the manufacturer]... I'm not happy with the answer that I was provided with. I have taken other products from this company and it's not that I don't feel like they manufacture unsafe products, I just don't particularly agree with how they handled this particular situation. (Consumer 8)

A related barrier to reporting directly to Health Canada's ADR reporting system was that participants did not always know that reporting their suspected ADR to Health Canada was an option. Typical of this was the response of one respondent who stated he was not familiar with the process for reporting. He went on to add, "... They should make people more aware of it". (Consumer 3)

For participants who knew that reporting to Health Canada was an option, another barrier was the perceived complexity of the reporting process online. For example, the following participant knew that online reporting to Health Canada was encouraged but still had trouble navigating through the system.

I was trying to follow the different links [to report online] but there were so many layers, maybe three or four layers, and you had to consent, you know, this is for information purposes and it wasn't very easy to do, I have to say. (Consumer 2)

Another barrier to reporting, described by several participants, was the fear that the implicated product would be withdrawn from the market, which would limit or prevent access to it in the future.

It is the only product that I've found effective for my health condition. It is something that I feel when used properly it doesn't cause an adverse reaction. I think that [reporting] jeopardizes the status of products that may be required for certain health conditions. (Consumer 7)

It's totally inconsistent as to how I can even access it, which is a problem... hence my concerns about it being pulled from the market. (Consumer 6)

## Discussion

Participants in this study had all experienced what they perceived to be suspected ADRs associated with NHPs. The products were usually self-prescribed and most participants decided either to handle the situation on their own or, less commonly, to discuss it with someone with whom they felt comfortable. The trusted person was typically thought to be someone working in a health food store or the company that had manufactured the implicated product, rather than a conventional healthcare provider. Participants either reported to the manufacturer directly or via the health food store. Other participants who did not report said that if their concerns were more pressing, they would feel more comfortable contacting the health food store personnel rather than their conventional health provider. Participants' responses to experiencing NHP-related suspected ADRs were based partly on their perceptions of the severity and seriousness of the reactions, reflecting the importance of individual perceptions in reporting ADRs.

Participants identified experiences as suspected ADRs and linked them to use of particular NHPs. In many cases, the processes participants used to do this were systematic and to some extent reflected some of the strategies used by healthcare professionals in their response to a patient reporting to them a suspected ADR, as well as the evaluative processes used by pharmacovigilance experts to assess causality. Despite consumers' lack of background medication knowledge, this process was similar to protocols used by health professionals to investigate potential ADRs i.e., the likelihood that symptoms are caused by a specific product. For example, participants considered the temporal relationship between product use and symptom appearance, the effects of discontinuing the product (de-challenge), re-challenging with the product, and other changes in their medication regimen [18,19].

In many cases, participants explained that they did not report the NHP-related suspected ADRs to Health Canada's ADR reporting system because they were unaware that such things could be reported and/or they did not know where to report. Most national ADR reporting schemes do not allow reports direct from consumers because of concerns about low-quality reports (e.g., missing important clinical information) and about the potential for obscuring important signals due to large numbers of reports [19,20]. Although Health Canada has always accepted direct reporting of suspected ADRs by consumers, this is not widely advertised and consumer reports are adjudicated separately from reports submitted by healthcare providers. In Canada, consumer reports are usually used to clarify or provide additional support for suspected ADRs (or signals) raised from healthcare provider reports.

Our data suggest that a lack of awareness about the opportunity for direct consumer reporting is limiting reporting by consumers. However, fear about what happens after submission of a consumer ADR report and distrust of Health Canada's regulatory response and its impact on the NHP market are also barriers for consumers. These concerns may explain why participants appeared to prefer reporting an NHP-related suspected ADR to someone they trusted - in this case health food store personnel and NHP companies.

A key finding was that the relationship between consumers and their service providers can act as a barrier or facilitator to reporting of suspected ADRs. Other research suggests that participants perceive high quality healthcare to include recognition of their status as informed patients and acknowledgement of their personal worth [21,22]. In the present study, participants felt that their physicians or pharmacists would ignore their concerns or question their values and beliefs in NHPs. This type of response negatively impacts the participants' abilities to maintain open and honest relationships with their healthcare providers. Participants who did not trust their conventional practitioners to respect their NHP-related choices sought out other advisors; in this study, that often included health food store staff. This is problematic though, as Canadian health food stores are not regulated, there are no common standards for employee training and the quality of advice given may be variable. In addition, health food store staff generally have no knowledge of the ADR reporting systems [23-25] and, therefore, reporting suspected ADRs to health food store staff is not likely to lead to the report being captured by the passive surveillance system.

The finding that some consumers report adverse events to manufacturers is important in that it provides another avenue through which Health Canada can access adverse event information. Manufacturers are responsible for reporting this information back to Health Canada. It is therefore important to ensure that this avenue of communication is robust. More research is needed in this area because NHPs are currently lightly regulated compared to pharmaceuticals. Competing interests and communication systems within the manufacturing industry may cause further complications to safety monitoring.

Under-reporting to passive surveillance systems is not new [12,26-29], however NHP ADR monitoring has additional complexities beyond the concerns of pharmaceutical post marketing surveillance. In particular, the consumer's comfort level with their health care providers impacts on their inclination to report use and experience of ADRs associated with NHPs. In addition, the chance of consumers contacting health food stores and manufacturers has not been discussed in pharmaceutical ADR literature. These issues need to be further examined in order to improve safe use of these products.

## Limitations

The study has several limitations that merit explanation. Interviews were only conducted with people who had already decided that their suspected ADR was linked to their use of a particular NHP. We were unable to explore the decision-making processes of individuals who experienced suspected ADR(s) while taking NHPs, but who had decided that these products were not associated with their ADR(s). In addition, due to the difficulty in recruiting participants, there was no time limit related to when the ADR was experienced. Some participants had experienced their reaction a few years before the interview. Based on the high level of detail and the emotional impact of the reactions experienced more than 3 months prior to the interview, these were significant events in the participants' lives that they remembered clearly, although recall bias cannot be completely discounted. Also, the demographic profile of the study participants is not representative of that of the Canadian population: our sample was highly educated and, in many cases, very familiar with NHPs, so further research with less well educated and less knowledgeable NHPs users (assuming they could be recruited) may be warranted. Given the high levels of education, it is likely that our sample was more knowledgeable about NHPs and options for reporting than those we were unable to interview. Despite the limited sample, the behaviours of the participants highlight important deficiencies in ADR reporting in a knowledgeable population [30]. The diversity of the products used and types of suspected ADRs enabled analysis of a range of experiences and saturation in the key themes as one would expect in this type of exploratory study.

## Conclusion

Consumers' descriptions of their NHP-related ADR experiences and reporting behaviours highlight several issues related to the current safety monitoring system. Few consumers know about the reporting opportunities, even fewer report directly via the Health Canada vigilance system. In addition, consumers fail to tell their physicians or pharmacists about these suspected reactions, which means that the current passive surveillance system is not capturing the majority of NHP-related suspected ADRs. Our study provides some explanations as to why this is occurring.

Consumers in our study who have experienced an NHP-related suspected ADR are unlikely to discuss their experiences with a health professional often because they self-prescribed the product and thus feel a need to "take responsibility" for the decision to use the NHP. This is compounded by consumers' perceptions that conventional healthcare providers will not understand their decisions to use NHPs and thus fear that they will not be supported to address the NHP-related suspected ADR. This study highlights the need for health professionals to be open-minded about their patients' choices regarding NHPs and the importance of developing good communication around NHPs so that patients will be comfortable sharing suspected ADR experiences. While under-reporting is a well established challenge of passive surveillance systems, the problems associated with NHP ADR monitoring are more complex than those associated with pharmaceutical drugs.

This study has identified several issues with implications for NHP safety monitoring. First, healthcare providers must be open to discussing NHPs with their patients, recognizing that many patients (likely at least half) may be using these products. Previous research suggests that initiation of NHP dialogue should occur at the first stages of patient contact, perhaps during regular medicine history taking [31]. It is also important to recognize that while disclosure of NHP use is essential, successful communication does not mean that healthcare practitioners have to approve of patients' choices [32]. In the case of NHP-related ADRs, communication of this type seems essential to enabling a trusting relationship. Our findings suggest that only when healthcare practitioners are seen as sources of information and support will consumers disclose their concerns when experiencing an NHP-related ADR.

This study also found that consumers are generally unaware that they are able to report directly to Health Canada and therefore, there is a need for initiatives to raise awareness among NHP users of this avenue for reporting suspected NHP ADRs. Spontaneous reporting systems are at present the mainstay of detecting signals of safety concerns associated with NHPs [12]. If suspected ADRs associated with NHPs do not reach the system, either through direct patient reporting or through reporting from healthcare professionals, then the detection of safety concerns may be missed or delayed. This has important implications for protection of the public health.

## Competing interests

The authors declare that they have no competing interests.

## Authors' contributions

RW and HB conceptualized and designed the study. RW carried out the interviews, coded the transcripts and wrote the first draft of the paper. HB supervised the project, coded the transcripts and edited the drafts of the paper. JB, ZA, SW and GRB participated in the design of the study and commented on drafts of the paper. All authors read and approved the final manuscript.

## Pre-publication history

The pre-publication history for this paper can be accessed here:

http://www.biomedcentral.com/1472-6882/10/8/prepub
